# Endocannabinoids and Heart Rate Variability Alterations after Exposure to Prolonged Intensive Physical Exercise of the Hellenic Navy SEALs

**DOI:** 10.3390/ijerph19010028

**Published:** 2021-12-21

**Authors:** Stamatis Mourtakos, Georgia Vassiliou, Konstantinos Kontoangelos, Anastassios Philippou, Elias Tzavellas, José Francisco Tornero-Aguilera, Vicente Javier Clemente-Suárez, Charalabos Papageorgiou, Labros S. Sidossis, Christos Papageorgiou

**Affiliations:** 1 Department of Psychiatry, National and Kapodistrian University of Athens, 115 27 Athens, Greece; geovassiliou@med.uoa.gr (G.V.); kontoangel@med.uoa.gr (K.K.); etzavell@med.uoa.gr (E.T.); chpapag@med.uoa.gr (C.P.); 2 Department of Physiology, Medical School, National and Kapodistrian University of Athens, 115 27 Athens, Greece; tfilipou@med.uoa.gr; 3 Neurosciences and Precision Medicine Research Institute “Costas Stefanis”, University Mental Health, 115 27 Athens, Greece; 4 Faculty of Sport Sciences, Universidad Europea de Madrid, 28670 Villaviciosa de Odón, Spain; josefrancisco.tornero@universidadeuropea.es (J.F.T.-A.); vctxente@yahoo.es (V.J.C.-S.); 5 Grupo de Investigación en Cultura, Educación y Sociedad, Universidad de la Costa, Barranquilla 080002, Colombia; 6 Department of Nutrition and Dietetics, Harokopio University of Athens, 115 27 Athens, Greece; lsidossis@kines.rutgers.edu; 7 Department of Kinesiology and Health, Division of Life Sciences, Rutgers University, New Brunswick, NJ 08901, USA; 8 251 Air Force General Hospital, 115 27 Athens, Greece; chrispapageorgio@gmail.com

**Keywords:** endocannabinoids, heart rate variability, acute stress, physical exercise, special forces

## Abstract

Background: Recent research indicates that both endocannabinoids (eCB) and heart rate variability (HRV) are associated with stress-induced experiences. However, these underlying mechanisms are not elucidated. The present study aims to investigate whether exposure to acute and chronic stress conditions can give rise to measurable changes, both to the peripheral eCB ligands and HRV. Methods: Thirteen candidates under intense preparation for their enlistment in the Hellenic Navy SEALs (HNS) participated in the study. All subjects underwent mental state examination, while HRV variables in time and frequency domain recordings were acquired. Furthermore, at baseline and 30 days after prolonged and intensive physical exercise, hair was collected to measure eCB ligands, such as anandamide (AEA), 2-arachidonoylglycerol (2-AG), and the *N*-acyl ethanolamine (NAE) molecules: palmitoylethanolamide (PEA) and oleoylethanolamide (OEA). Results: Comparing basal hair concentrations of eCB ligands before and after intense physical exercise, we found that AEA, PEA, and OEA were notably increased, whereas no differences were observed regarding the ligand 2-AG. Furthermore, there were observed associations between the concentrations of peripheral eCB ligands, both at baseline and after the prolonged physical exercise and the time and frequency domains of HRV. Conclusions: These findings suggest that endocannabinoid–HRV interrelations might share a short-term, and long-term adaptability of the changes in self-regulation associated with stress. Further studies will be required to determine the validity of peripheral eCB signaling and HRV as a biomarker for different aspects of the stress response.

## 1. Introduction

The endocannabinoid (eCB) system comprises of endogenous cannabinoid receptors CB_1_ and CB_2_, and endocannabinoid ligands. The CB_1_ receptors modulate appetitive behavior and mental functions as well as sensorimotor events, especially during pain management [1], while CB_2_ receptors are associated with the control of immune procedures [2]. However, most of the systematic studies have focused on the ligands of eCB, anandamide (AEA) and 2-arachidonoylglycerol (2-AG), which both connect to CB_1_ and CB_2_ receptors. Other ligands of eCB that have attracted the research interest are the N-acyl-ethanolamides (NAE), since they include palmitoylethanolamide (PEA) and oleoylethanolamide (OEA). In this line, PEA has been conceived as an anti-inflammatory mediator displaying pain-relieving features [3]. OEA is believed to be an agent showing appetite controlling properties by inducing the sense of satiety, contributing to the reduction in food intake [4]. In general terms, eCB ligands are produced “on-demand from cell membrane components (glycerophospholipids) in the brain and periphery” [5]. That is why recent lines of research have focused on the eCB system due to its function in stress control.

In this line, the experience of stress, not only acute but also chronic, usually leads to a two-way alteration of AEA and 2-AG, with AEA being decreased and 2-AG being enhanced [6]. These outcomes were ascribed in a bidirectional influence of these eCB ligands on the hypothalamic–pituitary–adrenal (HPA) axis. Specifically, it is thought that AEA triggers the HPA, escalating anxiety conduct and evolving sadness and increased pain sensitivity. On the other hand, it is believed that 2-AG ligand protects the effects of stress “by the termination of stress-induced HPA axis activation and promoting habituation to stress” [6]. In this context, stress-induced experiences lead to reduced OEA and PEA levels in hairs. In particular, lower hair PEA and OEA levels have been reported among patients with PTSD [7] and childhood maltreatment [8]. The benefits of hair investigations are characterized by their consistent validity and reliability. However, these analyses allow a trait picture of the subject matter instead of a state picture, which is permitted when using body fluids. [9]

Further studies in humans and animals showed that eCB is known to modify cardiovascular events such as heart rate, vascular tone, and blood pressure. As mentioned above, the ligands of the eCB are present not only in the nervous system (central and autonomous) but also in the periphery such as the myocardium and vasculature [5]. In addition, it has been shown that the administration of exogenous eCB (i.e., phytocannabinoids) in studies of humans and animals, exerts a chronotropic influence on heart rate [10,11,12]. At this point, it is useful to bring into mind that heart rate variability (HRV) is sensitive to changes in autonomic nervous system (ANS) activity (i.e., changes in the sympathetic nervous system (SNS) and parasympathetic nervous system (PNS)) associated with stress [12,13,14]. Neuroimaging studies suggested that HRV may be linked to cortical regions (e.g., the ventromedial prefrontal cortex) that are involved in stressful situation appraisal. Recent psychophysiological studies offer evidence indicating that heart rate variability (HRV) is affected by stress. In particular, brain imaging studies showed that HRV is correlated with brain networks that are responsible for the judgment of stressful situations (e.g., the ventromedial prefrontal cortex). This perspective supports the utilization of HRV for the objective investigation of stress-induced events [12]. In confirmation of this view, increased occupational stress was associated with reduced HRV [15]. Thus, it has been proposed that HRV might be a biomarker for emotion regulation. Its capacity has been extended, making HRV a surrogate parameter for the more general top-down ability to self-regulate, which is again coupled to the vagus nerve to the heart [16,17].

Taking into account the above considerations, the present study aims to evaluate the peripheral ligands of AEA and 2-AG, and the NAE molecules PEA and OEA, under basal conditions, immediately following a cognitive and emotional Stroop test, assigned to be recorded simultaneously with HRV and 30 days after prolonged and intensive physical exercise, in a group of military candidates of the Hellenic Navy Seal Forces. The main purpose was to investigate whether exposure to prolonged physical exercise (chronic stress) can lead to measurable changes in the peripheral EC ligands as they are assessed by hair analyses, with the hypothesis that the AEA would exhibit reduced levels whereas the 2-AG ligand, OEA, and PEA would exhibit increased levels. A secondary objective of this study was to establish whether there was a link between HRV and eCB ligands induced both in acute and chronic stress conditions.

## 2. Materials and Methods

### 2.1. Ethical Approval

The study was approved by the Ethics Committee of Harokopio University of Athens and was conducted in accordance with the Declaration of Helsinki. These studies were conducted after review and approval by the Hellenic Navy General Staff.

### 2.2. Subjects

For this study, we recruited all the candidates (*n* = 80) of the Basic Underwater Demolition SEAL (BUD/S) training of the Hellenic Navy Special Operations Command that started on December 2018. Out of the 80 candidates that were first included in the training program, only 13 were able to complete “Hell Week” (age 24.6 ± 3.8 years, height 180.4 ± 1.7 cm, body mass 78.0 ± 12.7 kg, body mass index 24.4 ± 0.2). All the procedures conducted were in accordance with the Helsinki protocol and have the Institutional Ethic Committee approval (code 55/5-4-2017).

### 2.3. Exercise Training Program

Hellenic Navy (HN) Special Operations Command (SOC) is the elite unit of the HN Special Operations Forces (SOF) community. HN SOC’s mission is to conduct unconventional warfare and amphibious operations in and out of Greek national territory area as NATO’s member. This study was conducted right before the most demanding military training week (Hell Week) of the Basic Underwater Demolition School (BUD/S) of the Hellenic Navy Special Operations Command. During this period, candidates participate in five days of continuous training with mental and physical fatigue. Each candidate has no sleep during the entire week, stays wet almost all the time, walks and runs more than 300 km, and does physical training for more than 20 h per day while experiencing continuous psychological pressure to perform optimally.

### 2.4. Procedure

The study was conducted at the base of the Basic Underwater Demolition School (BUD/S) of the Hellenic Navy Special Operations Command. HSN was examined in two different periods: right before the beginning of the “Hell Week” and right after the “Hell Week”. Each participant entered the procedure separately. Upon entering, a wristband was placed on their wrist for psychophysiological measurement. Afterward, saliva and hair were extracted. The duration of the procedure was 10 min (±5 min).

### 2.5. Materials

#### 2.5.1. Psychometric Tools

*Symptoms Checklist 90 Revised (SCL-90R):* This is a self-report instrument, used as a screening tool for current mental state. It consists of 90 questions that describe psychological, behavioral, and somatic symptoms separated into nine sub-categories. These sub-categories are somatization, obsessive-compulsive, interpersonal vulnerability, depression, anxiety, hostility, phobic anxiety, paranoid ideation psychoticism. It is based on a 5-point Likert scale from 0 = not at all to 4 = very much. In addition to the score extracted for each sub-category, there are three more indices: the global severity index, the positive symptom distress index, and the positive symptom total. It has been standardized in Greek population [18].

#### 2.5.2. Psychophysiological Measurements:

In order to measure autonomic response, First Psychiatric Clinic of National and Kapodistrian University has developed a collaboration with Sentio Solutions Inc., which provides the Laboratory of Psychophysiology with a wristband called “FEEL”. FEEL’s technology makes it capable of measuring heart rate variability, electrodermal response, and temperature. Utilizing four built-in sensors in the wristband, FEEL collects information on the aforementioned biosignals and analyzes them using the most modern technological methods. Advanced technologies related to artificial intelligence and signal process algorithms are in a position to track down emotional reactions while participants are wearing the wristband and executing various tasks. Heart rate is detected through a sensor that works as a plethysmograph. Changes in heart rate and heart rate variability are thought to indicate changes in participants’ emotional states. More specifically, regarding heart rate variability, it can give information for 77 different features, which include time domain (e.g., *mean HR*, *std HR*, *mean HRV*, *SDNN*, *mean first difference and mean second difference*) and frequency domain (e.g., *LF*, *MF*, *HF*, *LF/HF*, *total power content*), as well as non-linear and wavelet features.

**Figure 1:**
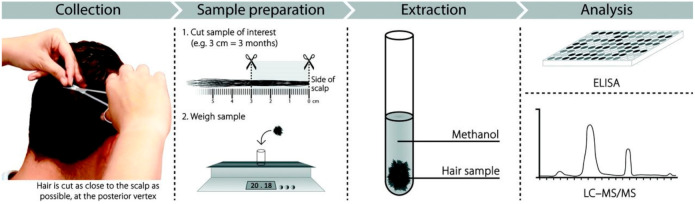
Hair sample collection and analysis.

*Hair sample collection:* Scalp hair collection is straightforward and easily performed in an outpatient setting (Figure 1). In accordance with guidelines published by the Society of Hair Testing, the hair sample is collected from the posterior vertex. Cortisol and endocannabinoids are extracted from 5–10 mg whole hair by methanol incubation and isopropanol washes, followed by a column switching strategy for on-line solid-phase extraction (SPE). The extracted substances are measured using ELISA or liquid chromatography-tandem–mass spectrometry (LC–MS/MS) on a Q-Trap mass spectrometer (ABMDS SCIEX, Concord, Ontario, Canada) according to SoHT and EWDTS guidelines [19,20,21,22,23,24].

#### 2.5.3. Statistical Analysis

Values are presented as absolute and relative frequencies for nominal variables concerning demographic data and as the mean and standard deviation for the continuous variables regarding the scales investigated in this work. Normality assumption was examined via Shapiro–Wilks test. Comparisons between before and after “Hell Week” as regards the endocannabinoid levels were performed by using parametric and non-parametric tests with a selected significance level of 5%. Specifically, two independent samples t-test was conducted for testing the equality of mean values between before and after endocannabinoid levels for normally distributed variables; in addition the Mann–Whitney test was conducted for testing the equality of median values for non-normally distributed variables. The effect size was evaluated by calculating Hedges’ g value and eta square. Data analysis was performed using the statistical software of IBM SPSS (Version 23) (IBM Corp, Armonk, NY, USA).

## 3. Results

Thirteen HNS were included in the analysis. Table 1 presents their demographic characteristics. Participants were between 19 and 29 years of age with a mean age of 24.63. No one had health problems or received medicine.

Regarding the psychometric evaluation of their mental state measured via SCL-90R, participants were found to be different in four sub-categories from the general population. More specifically, they had a statistically significantly higher mean level of somatization (t(9,10) = 2.334, *p* < 0.05) with a medium size effect Cohen’s d equal to 0.74 (Table 2). In addition, HNS had a statistically significantly lower mean level at the categories of interpersonal sensitivity (t(9,10) = −3.445, *p* < 0.01) with a size effect Cohen’s d equal to 1.09, hostility (t(9,10) = −4.614, *p* < 0.01) with a size effect Cohen’s d equal to 1.46, and paranoid ideation (t(9,10) = −2.290, *p* < 0.05) with medium size effect Cohen’s d equal to 0.72 (Table 2).

Endocannabinoids levels were measured before and after a month of an intensive physical stress condition of the same sample. Conducting a paired sample t-test, differences were found in three out of the four ligands of eCB (Table 3). More specifically, levels of AEA were statistically significantly higher after the intensive training (t(12) = −4.225, *p* < 0.01) with a size effect of Cohen’s d equal to 1.17. Furthermore, levels of PEA were also statistically significantly higher after the month of physical stress (t(12) = −2.963, *p* < 0.05) with large size effect Cohen’s d equal to 0.82. Finally, levels of OEA were found to be statistically significantly higher in the after measurement (t(12) = −5.115, *p* < 0.001) with a size effect of Cohen’s d equal to 1.42.

No statistical difference was observed in any of the HRV features before and after a month of an intensive physical stress condition. Correlations were conducted between the ligands of eCB and the time and frequency domain features of HRV. Regarding the time domain features of HRV, a strong positive correlation was found between mean HR and AEA (r(13) = 0.934, *p* < 0.05) (Table 4). Furthermore, mean HRV was found to be strongly correlated in a positive way with PEA (r(13) = 0.902, *p* < 0.05) (Table 4). Regarding frequency domain, a strong negative correlation was found between low-frequency (LF) HRV and PEA (r(13) = −0.853, *p* < 0.01) (Table 4). There were no statistically significant associations between high-frequency HRV and EC ligands.

Finally, we conducted correlations between the mean difference of the ligands of eCB before and after the month of intensive physical stress, and time and frequency domain features of HRV before the training (Table 5). A moderate positive correlation was found between the mean difference of OEA and mean HR (r(13) = 0.683, *p* < 0.05), and a moderate negative correlation was found between the mean difference of OEA and mean HRV (r(13) = −0.640, *p* < 0.05). Furthermore, the mean difference of PEA was found to be correlated both with LF HRV (r(13) = −0.709, *p* < 0.05) and HF HRV (r(13) = 0.751, *p* < 0.05).

## 4. Discussion

Special Operations Forces are an elite subset of the military population who are expertly trained to cultivate and deliver specialized warfare capabilities beyond those of standard military forces. During the military training week analyzed (Hell Week) they experienced extreme psychophysiological stress, physical pain, and fatigue. The results obtained suggest that the ligands AEA, PEA, and OEA of eCB exhibited statistically significant augmentation after a month of prolonged exposure to intensive stress conditions, whereas no differences were observed regarding the ligand 2-AG. Furthermore, associations were obtained between the time and frequency domains of HRV, elicited during the initial phase of the study, and peripheral ligands of eCB. In particular, positive associations between the time domain of the HRV and AEA and PEA ligands of eCB regarding the Mean HRV (r = 0.934, *p* = 0.020) and Mean HR (r = 0.902, *p* = 0.036), respectively, were found. Noticeable also were the negative associations between the low frequency (LF) of the HRV and the PEA ligands (r = −0. 853, *p* = 0.002).

Additionally, there were correlations between the time domain of the HRV and the change in OEA ligands (basal line minus final levels) where the mean HR exhibited positive associations, whereas the mean HRV exhibited negative associations (r = 0.683, *p* = 0.029 & r = −0.640, *p* = 0.046, respectively). Finally, the LF of the HRV exhibited a negative relationship with the changes in the PEA ligands (basal line minus final levels) (r = −0.709, *p* = 0.022), while the high frequency (HF) of the HRV exhibited a positive relationship with the PEA ligands (r = 0.751, *p* = 0.012). The concentrations of AEA, PEA, and OEA, but not 2-AG, exhibited augmentation after exposure to the chronic stress in comparison to basal levels.

While the validity of the reduction in AEA patterns as an index of stress-induced regulation of eCB signaling appears to be confirmed by a wide array of stress paradigms [6,25,26,27,28], there are a few studies that have pointed to a different direction [29,30]. It has been argued that increased AEA levels could be manifested in stressful situations where a painful component is present. This paradox may be due to the activation of neural pathways that prepare AEA to adjust to diverse aspects of the stress response, such as pain [6]. Furthermore, the obtained increased levels of OEA and PEA ligands of eCB appear to be compatible with other reports. PEA has been conceived as an anti-inflammatory mediator displaying also pain-relieving features [3], and OEA is believed to be an agent showing appetite controlling properties by inducing the sense of satiety, thus contributing to a reduction in food intake [4].

The results concerning the 2-AG ligands of eCB are partially consistent with preclinical and clinical studies which have demonstrated that exposure to prolonged stress evoked increased 2-AG. As a matter of fact, the stress-induced 2-AG level is transient as has been shown in related studies. In particular, it was reported that in a condition of constant stress, on the tenth day the concentration of 2-AG in the amygdala was greater 20 min after the start of stress and had come back to the initial values after 60 min [27], remaining at these levels for 24 h after the stress finished [25]. This dispute might be better understood by considering the view that the AEA ligand of eCB indexes the “tonic” signaling mean of it, whereas the 2-AG embodies the “phasic” signaling agent of eCB, mediating numerous procedures of “synaptic plasticity” [31,32].

The observed positive association between the mean HRV and AEA at baseline could be better understood by considering the biological meaning of both HRV and AEA. Indeed, high HRV is linked with controlling and homeostatic autonomic nervous system operations, which increase the body’s aptitude to manage the distress conditions [12], whereas stress-induced events reduce AEA levels [6]. Though there is evidence indicating a negative association between AEA and stress, the principal processes eliciting this phenomenon are debatable. One possible account for this association is that enhanced AEA levels trigger effects of wellbeing by activating corticolimbic brain networks responsible for reward [32,33,34].

The negative association between low-frequency LF and PEA ligands at baseline might be considered both in the overall autonomic context and in association with the endocannabinoid signaling induced by environmental challenges. Indeed, increased LF of HRV indicates a low parasympathetic activity of HRV, which is interrelated with the reduction in the threat perception mediated by the brain networks responsible for the evaluation of stressful events [12]. On the other hand, PEA is believed to be implicated in endogenous protecting processes that are mobilized by the stimulation of inflammatory or nociceptive procedures [35].

The positive associations between heart rate and both 2-AG and PEA at baseline might be conceived as taking into account the possible involvement of eCB ligands in the cardiovascular modulation during the stress exposure. In this framework, heart rate is raised up when the subjects are exposed to stress due to the requirement for increased metabolic activity, whereas 2-AG and PEA both participate in the regulation of nociception and coordination of energy utilization due to the stress-induced challenge [26,36]. However, HRV is considered a consistent index of the ANS operation induced by stress. The HF is regarded as a mirror of the PNS function, whereas LF signifies the action of the SNS [12,37]. The obtained associations between the peripheral OEA ligands of eCB and time domain of the HRV at baseline (the heart rate and HRV), as well as the peripheral PEA ligands eCB and the frequency domain of the HRV at baseline (LF and HF), suggests that peripheral eCB ligands believed to be involved in anti-inflammatory and anorexigenic effects evoked by prolonged stress exercise might be related to emotional stress-related changes in the SNS and PNS. These results support evidence based on preclinical studies, indicating that elements of the peripheral eCB system reflect central dysfunctions of eCB [35,37].

Finally, the large contextual stressor of the “hell week”, as well as in other SOF actions [37,38,39,40,41], required from operators a psychological profile that differs from the normal population in order to succeed under these this extreme tests. This was manifested in the significant differences in the somatization, interpersonal sensitivity, hostility, and paranoid ideation variables of SLC-90 compared with the general population. This specific psychological profile, as well as a highly adaptative autonomic modulation, allow operators to be able to overcome the high demands of these highly operational units [40,42,43,44].

This study presents some limitations, such as the lack of a control group or the inclusion of other HRV domains as non-linear analysis, alpha 1 or alpha 2 variables, or fractal dimensions.

## 5. Conclusions

Taken together, these data may give evidence of the role of peripheral endocannabinoid signaling during ongoing levels of stress that influence the autonomic activity involved in stress and support the utility of simultaneous assessments of both the peripheral eCBs and HRV as objective markers of stress-related conditions. Furthermore, prolonged stress induced by physical exercise activates peripheral endocannabinoids, which could contribute to the replenishing of energy stores and also to the pain relieving and mood-modulating effects of exercise.

## Figures and Tables

**Table 1:** Demographic characteristics of participating subjects.

Demographic Characteristics	HN-SEALs
Number of participants	13
Age (years)	24.63 ± 3.81
Health Problem (No)	13 (100%)
Receiving medicine (No)	13 (100%)

**Table 2:** One sample t-test for equality of SCL-90 mean values between HNS and general population.

	Sample Mean (±sd)	Population Means (±sd)	One Sample *t*-test	Effect Size Cohen’s d
Somatization	13.8 (±8.51)	7.52 (±6.35)	2.334 *	0.74
Interpersonal sensitivity	3.6 (±3.86)	7.81 (±6.04)	−3.445 **	1.09
Hostility	2.3 (±2.11)	5.38 (±4.78)	−4.614 **	1.46
Paranoid Ideation	3.3 (±3.46)	5.81 (±3.64)	−2.290 *	0.72

* *p* < 0.05, ** *p* < 0.01.

**Table 3:** Paired sample t-test for equality of AEA, AG2, PEA, and OEA mean values before and after intensive physical stress.

	Mean (±sd) before	Mean (±sd) after	Paired Sample *t*-test	Effect Size Cohen’s d
AEA	3.46 (±0.46)	3.90 (±0.59)	−4.225 **	−1.17
AG2	72.55 (±5.80)	72.52 (±5.96)	0.083	0.23
PEA	1453.94 (±150.72)	1633.64 (±195.94)	−2.963 *	−0.82
OEA	1972.49 (±39.60)	2138.19 (±137.81)	−5.115 ***	−1.42

* *p* < 0.05, ** *p* < 0.01, *** *p* < 0.001.

**Table 4:** Correlations between time and frequency domain of HRV and ligands of eCB.

	Mean HR	Mean HRV	LF HRV
AEA	−0.756	0.934 *	−0.037
PEA	0.902 *	−0.056	0.853 **

* *p* < 0.05, ** *p* < 0.01.

**Table 5:** Correlations between mean difference of ligands of eCB and time and frequency domains of HRV.

	Mean HR	Mean HRV	LF HRV	HF HRV
Mean difference PEA	0.475	−0.559	0.751 *	−0.709 *
Mean difference OEA	0.683*	−0.640 *	0.182	−0.277

* *p* < 0.05.

## Data Availability

All data are in the text.
